# Electroactive 4D Porous Scaffold Based on Conducting Polymer as a Responsive and Dynamic *In Vitro* Cell Culture Platform

**DOI:** 10.1021/acsami.3c16686

**Published:** 2024-01-26

**Authors:** Franziska Hahn, Ana Ferrandez-Montero, Mélodie Queri, Cédric Vancaeyzeele, Cédric Plesse, Rémy Agniel, Johanne Leroy-Dudal

**Affiliations:** † Equipe de Recherche sur les Relations Matrice Extracellulaire-Cellules (ERRMECe), Groupe Matrice Extracellulaire et Physiopathologie (MECuP), I-Mat, CY Cergy Paris Université, 95000 Neuville sur Oise, France; ‡ Laboratoire de Physicochimie des Polymères et des Interfaces (LPPI), I-Mat, CY Cergy Paris Université, 95000 Neuville sur Oise, France; § Instituto de Ceramica y Vidrio (ICV), CSIC, Campus Cantoblanco, Kelsen 5., 28049 Madrid, Spain

**Keywords:** engineered cell microenvironment, 4D scaffolds, responsive cell culture platform, polyHIPE, PEDOT, electronic conducting polymers, *in situ* cell stimulation

## Abstract

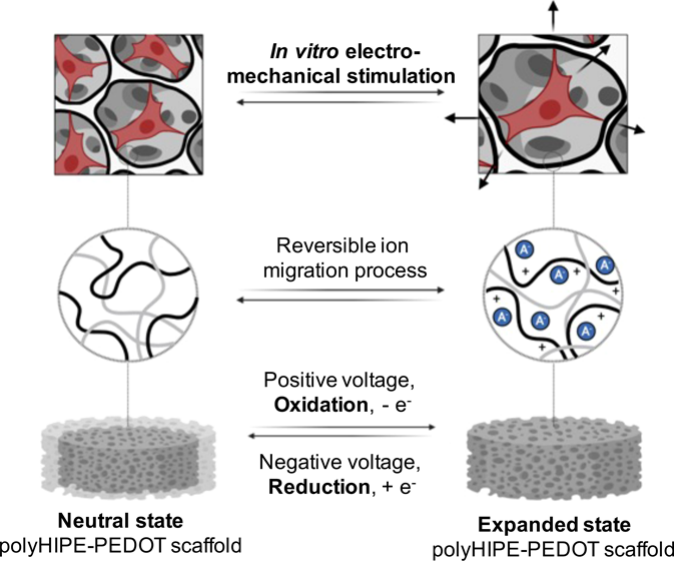

*In vivo*, cells reside in a 3D porous and dynamic microenvironment. It provides biochemical and biophysical cues that regulate cell behavior in physiological and pathological processes. In the context of fundamental cell biology research, tissue engineering, and cell-based drug screening systems, a challenge is to develop relevant *in vitro* models that could integrate the dynamic properties of the cell microenvironment. Taking advantage of the promising high internal phase emulsion templating, we here designed a polyHIPE scaffold with a wide interconnected porosity and functionalized its internal 3D surface with a thin layer of electroactive conducting polymer poly(3,4-ethylenedioxythiophene) (PEDOT) to turn it into a 4D electroresponsive scaffold. The resulting scaffold was cytocompatible with fibroblasts, supported cellular infiltration, and hosted cells, which display a 3D spreading morphology. It demonstrated robust actuation in ion- and protein-rich complex culture media, and its electroresponsiveness was not altered by fibroblast colonization. Thanks to customized electrochemical stimulation setups, the electromechanical response of the polyHIPE/PEDOT scaffolds was characterized *in situ* under a confocal microscope and showed 10% reversible volume variations. Finally, the setups were used to monitor in real time and *in situ* fibroblasts cultured into the polyHIPE/PEDOT scaffold during several cycles of electromechanical stimuli. Thus, we demonstrated the proof of concept of this tunable scaffold as a tool for future 4D cell culture and mechanobiology studies.

## Introduction

*In vivo*, cells reside in a complex and dynamic 3D microenvironment, providing both structural and functional support.^1,2^ The cell microenvironment includes a wide variety of biochemical and biophysical cues that orchestrate cell adhesion, proliferation, differentiation, and migration.^3^ In turn, the properties of the cell microenvironment constantly change under the influence of cells. Both this dynamic nature and the reciprocal interactions between cells and their environment regulate cell behavior during physiological and pathological processes.^2,4^ Regarding basic approaches to cell biology studies as well as the development of cell-based drug screening systems or scaffolds for tissue engineering, a major challenge is to develop physiopathologically relevant *in vitro* models that could integrate dynamics of the cell microenvironment.^4−6^

In this way, traditional 2D *in vitro* cell culture on plastic or flat glass substrates has rapidly evolved over the last two decades toward 3D culture models.^7,8^ 3D models allow cells to exhibit a more *in vivo*-related phenotype. The importance of matrix dimensionality (3D *vs* 2D) was particularly documented for fibroblasts, the major cells of connective tissue, which synthesize, organize, and maintain extracellular matrix homeostasis while contributing in response to injury.^9,10^ In 3D matrices, fibroblasts secrete more bioactive molecules and exhibit a bipolar or stellate shape, in contrast to the thin, flat, and extended morphology they display in 2D.

Natural and synthetic scaffold-based systems are extensively used to provide *in vitro*-appropriate 3D environments for hosting cells. The interest in synthetic scaffolds lies in the great possibilities to control and tune their mechanical properties and to be produced in a reproducible manner.^11,12^ Recent studies have shown that a porous architecture, with interconnected pores around 50–100 μm, is a key requirement to use scaffolds as 3D cell culture substrates due to the possibility of mass transport, nutrient diffusion, and cell ingrowth.^13^ Among the various techniques providing the incorporation of porosity into a polymer-based scaffold, such as particle leaching, gas foaming, ice templating, and others, high internal phase emulsion templating allows the generation of fully interconnected emulsion-templated porous polymer scaffolds. It is referred to as polymerized high internal phase emulsions (polyHIPEs). These latest 3D porous interconnected scaffolds are promising for cell hosting.^12,14−19^ Besides, some inert (*i.e*., unfunctionalized) 3D porous scaffolds of polyHIPE are already commercially available for routine 3D cell culture, such as Alvetex, a polystyrene-based scaffold suitable for a broad range of cell types and cell investigations.^16,20^ The main advantages of polyHIPE are their wide range of composition and their highly controllable interconnected porosity.^21^ However, despite the well-documented cytocompatibility of polyHIPE scaffolds, chemical modification or biofunctionalization of the surface is often required to enhance cell attachment and proliferation.^16^

In a constant effort to better mimic the cell environment *in vitro*, mechanical stimulation devices were introduced within 3D scaffolds to stimulate cells through deformation of the material.^1,22^ Compression, tension, or shear stresses trigger mechanotransduction within cells, a process in which the mechanical input is converted into a biological response. Nevertheless, most of these devices rely on applying external deformations to passive scaffolds and they fail to properly mimic the dynamic properties of the *in vivo* environment. Therefore, the use of stimuli-responsive materials as an active component of the 3D scaffolds can bridge the gap for recapitulating *in vivo* dynamics, adding a dynamic fourth dimension to the 3D cell culture.^2,23^ This field, the so-called *in vitro* 4D biology, while still in its early stages, is an attractive way to design 3D cell culture platforms with dynamic properties.^2^ For example, cell interactions with material or scaffold geometry could be dynamically manipulated by photoirradiation.^24,25^ However, these devices often lack reversibility. Thus, the field could be enlarged by the design of new cell culture platforms that would integrate 3D structure, tunable mechanical properties, and an extracellular matrix-like neighborhood to progress toward 4D dynamic systems.^2,26^

Electronic conducting polymers (ECPs), such as poly(3,4-ethylenedioxythiophene) (PEDOT), are promising candidates for the development of 4D-responsive materials thanks to their electrical conductivity and their ability to present reversible volume changes upon low-voltage electrochemical stimulation when immersed in electrolytic solution.^27^ Additionally, PEDOT exhibits high biocompatibility with various cell types, such as fibroblasts, neurons, cardiomyocytes, or stem cells.^28−31^ It also promotes cell adhesion and proliferation and tissue regeneration processes.^32,33^ Recently, we described a new method to produce electroresponsive 4D materials, relying on the functionalization of a passive 3D polyHIPE structure with PEDOT as the electroactive conducting polymer.^34^ When these materials were stimulated under low voltage (<1 V) in phosphate buffer saline (PBS), a reversible volumetric variation of 10% was obtained, demonstrating their electroresponsive 4D nature.

In this work, we explored the use of a 4D polyHIPE/PEDOT scaffold as an *in vitro* dynamic cell culture platform. The synthesized and functionalized scaffolds showed promising properties in terms of porosity, cytocompatibility, and biofunctionalization for cell culture. Our results show an early and fast colonization of the scaffolds by fibroblasts. Then, the ability of the polyHIPE/PEDOT scaffold to be actuated under electrostimulation, once incubated in physiological cell culture conditions, was evaluated. Finally, customized electrochemical stimulation setups were designed and implemented under a confocal microscope. With these systems, we demonstrated that a 4D dynamic cell culture can be carried out *in situ* and monitored in real time. Cell deformation induced by the scaffold actuation can be tracked and measured over time.

## Materials and Methods

### Fabrication of 4D PolyHIPE/PEDOT Scaffolds

The polyHIPE synthesis was adapted from procedures described by Cameron’s group.^14^ Briefly, the organic phase (20 vol % of the total emulsion) consists of the monomer poly(ethylene glycol) diacrylate (PEGDA, Mn = 700 g/mol, Sigma-Aldrich) and the cross-linker trimethylolpropane tris(3-mercaptopropianate) (TMPTMP, ≥95%, Sigma-Aldrich, 2/3 mol equiv of thiol functions *vs* acrylate functions), the surfactant (Hypermer B246-SO-(MV), Croda, 3 wt % of the organic phase), the photoinitiator (Darocur 1173, Sigma-Aldrich, 5 wt % of the organic phase), and dichloroethane (1,2-dichloroethane 99,8+%, extra pure, Thermo Scientific) (44.3 wt % PEGDA and 11.2 wt % TMPTMP and 44.5 wt % dichloroethane). The organic phase was emulsified with the aqueous phase (80 vol % of the total emulsion) added dropwise. The high internal phase emulsion (HIPE) was then cast between two glass plates (0.5 mm Teflon gasket), and polymerization was triggered by UV irradiation (Primarc UV Technology, Minicure, mercury vapor lamp, 100 W/cm, scan duration: 6 s). The water phase of the polyHIPE was removed by immersion in acetone overnight, while the poly(thioether) network synthesized within the organic phase stood as a porous monolith. The polyHIPE was further washed with a speed extractor (E-914, BUCHI) using dichloromethane and finally dried under vacuum at 60 °C overnight. The functionalization of polyHIPEs with PEDOT was performed according to a two-step procedure. First, 3,4-ethylenedioxythiophene monomer (EDOT, CLEVIOS MV2, Heraeus) was incorporated by vapor phase swelling within the walls of the polythioether network under a static vacuum at 50 °C. Depending on this step duration, a swelling ratio SR% of 120% of EDOT *vs* the initial mass of the polyHIPE was achieved. In a second step, the EDOT-swollen polyHIPEs were immersed in 1.5 M FeCl_3_ aqueous solution (iron(III) chloride anhydrous, Sigma-Aldrich) at 40 °C for 3 h to ensure the oxidative polymerization of EDOT into PEDOT. The polyHIPE/PEDOT material was washed using methanol and ethanol in order to remove excess iron chloride and residual EDOT. The material was finally dried under vacuum at 80 °C overnight.

### Morphological Characterization of 4D PolyHIPE/PEDOT Scaffolds

#### Scanning Electron Microscopy

The morphology of the polyHIPE and polyHIPE/PEDOT was analyzed by a field emission gun scanning electron microscope (SEM, GeminiSEM300, Zeiss) with an acceleration voltage of 2 keV under a high vacuum. Before acquisition, the materials were mounted directly on SEM stubs and sputtered with 4 nm of platinum (ACE600, Leica). During acquisition, secondary electrons were collected, and scan speed and line averaging were adjusted. Pore and interconnection diameters were analyzed by using image analysis of up to 100 voids and interconnections in several acquired micrographs.

#### Swelling of the PolyHIPE/PEDOT Scaffolds

Scaffolds were immersed in complete culture medium or PBS for 1 h at room temperature in order to determine the swelling ratio of the polyHIPE and polyHIPE/PEDOT samples.

#### Biofunctionalization with Fibronectin

Scaffolds were autoclaved (121 °C, 30 min) and biofunctionalized for 1 h at 37 °C by a coating of fluorescent fibronectin (50 μg/mL) from human blood plasma purified and modified according to published protocols.^35,36^

### Biological Characterization of 4D PolyHIPE/PEDOT Scaffolds

#### Scaffold Preparations for Cell Culture

The polyHIPE and polyHIPE/PEDOT scaffolds were cut into disk shapes with diameters compatible with 24- or 48-well microplates and then sterilized in an autoclave (121 °C, 30 min). Scaffolds were immersed in complete cell culture medium: Dulbecco’s modified Eagle’s medium (DMEM, Gibco) and 10% fetal calf serum (FCS, BioSera) were incubated at 37 °C overnight before the experiments. Supernatants from the scaffold immersion (preconditioned media) were stored at −80 °C for indirect cytotoxicity assays. Scaffolds were washed with PBS prior to cell seeding.

#### Fibroblast Cell Culture

Experiments were carried out with human skin fibroblasts (BJ CRL-2522, ATCC) and red fluorescent fibroblasts (red TTFLUOR HDF, Innoprot). Red TTFLUOR HDF cells were cultured on a poly-l-lysine-coated flask (2 μg/cm^2^, 0.01% poly-l-lysine solution, Sigma-Aldrich). Both cell lines were grown in complete cell culture medium at 37 °C with 5% CO_2_. Cells were dissociated with 0.25% of trypsin-EDTA (Gibco) once or twice a week. Cultures with preconditioned media were performed by incubating BJ cells for 48 h in complete cell culture medium preincubated with scaffolds for 24 h. Apart from routine cell culture, all experiments with both cell lines were then carried out without any coating of poly-l-lysine.

#### Cytotoxicity Assay

Cytotoxicity assays were carried out after 3, 24, 48, 72 h, and 7 days of BJ cell culture by assessing LDH activity in supernatants (preconditioned media) according to the manufacturer’s instructions (LDH Cytotoxicity Detection Kit, Takara). Controls consisting of cells cultured in classical 2D conditions and cell lysis with 1% v/v of Triton X-100 (T8787, Sigma-Aldrich) (positive control) were included. Cell experiments were performed in triplicate in at least two independent experiments.

#### Cell Colonization

Cell penetration analyses were performed on 0.8 cm diameter polyHIPE/PEDOT scaffolds seeded with 40,000 or 100,000 red TTFLUOR HDF and BJ cells:

(a) Cell penetration was followed after 24 h by laser scanning confocal microscope analysis (CLSM, LSM710, Zeiss and Stellaris 5, Leica) with a 40× oil immersion (NA 1.3) and a 20× dry (NA 0.8) objective. Topography was performed by collecting the reflection of the 633 nm laser, and the signal of the adherent cells was excited by a 561 nm laser. 3D fluorescence and topography images for polyHIPE/PEDOT scaffolds with cells were performed by optical sectioning. z-Stacks (sections 1 μm) were sequentially acquired. z-Stacks of confocal images could be visualized by ImageJ-FIJI as 3D visualization or as 2D images (z-projections) by using the maximum z-projection tool for each fluorescence channel.
(b) Cell penetration was followed by SEM after 3 h and 7 days. After washing in cacodylate buffer pH 7.3, polyHIPE/PEDOT scaffolds seeded with cells were dehydrated through graded ethanol series from 30 to 100% and critical point dried (CPD300, Leica). Scaffolds were cut into 1–2 mm thin slices for cross-sectional analysis or mounted directly on SEM stubs, sputtered with 4 nm of platinum (ACE600, Leica) and imaged using a SEM (GeminiSEM300, Carl Zeiss) with an acceleration voltage of 2 keV under high vacuum. Secondary electrons were collected. Scan speed and line averaging were adjusted during observation. Images were processed and colorized with MoutainsSEM (Digital Surf).
(c) Quantitative analysis of cell penetration for 3 h, 1, 3, or 7 days was carried out with a fluorescence wide-field microscope (DMi8 Thunder Imager, Leica) to acquire the cells’ fluorescence (excitation 555 nm). The medium was changed every second day. Before the fluorescence microscopy analysis, cells were fixated with 4% w/v of paraformaldehyde (PFA) for 10 min and placed in a glass-bottomed Petri dish. Images of the entire scaffold were acquired with a 10× objective. The bottom and top sides (cell seeding side) of the scaffold were imaged as z-stacks. Image analysis was done by ImageJ-FIJI. A z-projection was created from the z-stacks with the standard deviation calculation, thresholded, and the watershed function was used to separate touching cells into individual cells. The cell number for the bottom or top side of the scaffold was finally quantified using the analyze particle function. The colonization results were plotted *vs* the total cell number measured with the CyQUANT Cell Proliferation Assay Kit (Invitrogen) according to a modified version of the manufacturer’s instructions.

#### Immunofluorescence

100,000 BJ cells were cultured 24 h on polyHIPE/PEDOT scaffolds (0.8 cm in diameter), fixed with 4% w/v of PFA in PBS for 10 min, permeabilized with 0.1% v/v of Triton X-100, and incubated with 0.5% w/v of PBS-BSA. Cells were then incubated for 1 h at room temperature with mouse anti-α-tubulin (T9026, Sigma-Aldrich), vimentin (CBL202, Merck), or cellular fibronectin (ab6328, Abcam) antibodies diluted to 1/500. Samples were rinsed with PBS and incubated for 1 h with the secondary antibody coupled to Alexa Fluor488 (A11029, Invitrogen) diluted to 1/400. Nuclei were stained with DAPI (D9542, Sigma-Aldrich) diluted to 1 μg/mL. The topography and visualization of adherent cells were carried out by CLSM (LSM710, Zeiss and Stellaris 5, Leica) with a 40× oil immersion (NA = 1.3) and a 20× dry (NA = 0.8) objective. Z-stacks (sections 1 μm) were sequentially acquired with excitation wavelengths at 405 nm for DAPI and 488 nm for Alexa Fluor 488 secondary antibodies. Emission windows were set at 410–475 and 495–550 nm, respectively, for capturing the fluorescence emission of the dyes. Z-stacks of confocal images could be visualized by ImageJ-FIJI as 3D visualization or as 2D images (z-projections) by using the maximum z-projection tool for each fluorescence channel.

### Electrochemical Characterization and Actuation of 4D PolyHIPE/PEDOT Scaffolds

Electrochemical characterization was carried out by cyclic voltammetry (CV) at a scan rate of 10 mV/s from 0.8 V/-0.8 V for polyHIPE/PEDOT scaffolds of known dimensions using a VSP potentiometer (Interface1010E, Gamry instruments) in a classical three-electrode configuration in a degassed (Argon) PBS solution and complete cell culture medium (DMEM + 10% FCS). Ag wire was used as a pseudoreference (Ref.) and glassy carbon rod as a counter electrode (CE). The measured currents were normalized by the geometric surface area of the polyHIPE/PEDOT scaffold used as a working electrode (WE). Actuation of the polyHIPE/PEDOT scaffolds, monitored using a CLSM in reflection mode, was induced by alternating the potential at the scaffold from 0.8 to −0.4 V for 60 s at each oxidation and reduction step over 3 cycles in total using a VSP potentiometer (Interface1010E, Gamry Instruments). The confocal images were processed by using ImageJ-FIJI software. Their intensity values were falsely colored with a “rainbow” lookup table. With this color scheme, low-intensity pixels appeared in blue colors and high-intensity pixels in hot colors, such as orange and red. This approach aimed to enhance the visualization of the scaffold’s actuation.

### 4D Device for *In Situ* and Real-Time Monitoring of Cell Dynamics

*In situ* stimulation and real-time monitoring of red TTFLUOR HDF cells seeded on polyHIPE/PEDOT scaffolds with increased sizes (1.2 cm in diameter) were carried out with a customized setup in a two-electrode configuration. This setup consisted of a 3D-printed electrode holder and 2 glassy carbon rods serving as current collectors (3 mm in diameter) onto two polyHIPE/PEDOT scaffolds. Platinum/iridium (Pt80/Ir20) wires (0.1 mm in diameter) were connected to the electrodes and worked as a connection via crocodile clamps to a VSP potentiometer (Interface1010E, Gamry instruments). For the CAD model of the electrode holder, FreeCAD, an open-source 3D CAD software was used. The CAD model was later converted by the UltiMaker Cura software into a g-code file and printed by the extrusion-based 3D printer UltiMaker2. Smart Again was used as the printing material and is a mix of NYLON (PA6) and polyolefin polymers.

150,000 red TTFLUOR HDF cells were seeded on polyHIPE/PEDOT scaffolds (1.2 cm in diameter) and incubated for 3 h. Afterward, the cell-seeded scaffolds were transferred with the cell seeding side down to a glass-bottomed Petri dish, the two-electrode setup was placed on top of the scaffolds, fresh complete cell culture medium was added, and a potential difference (Δ*E*) of ±1.5 V was applied for 90 s at each oxidation and reduction step using a VSP potentiometer (Interface1010E, Gamry Instruments). The stimulation lasts 9 min (3 cycles in total). The connection of the scaffold to the electrodes was checked before the actuation measurements by CV at an Δ*E* of ±1.0 V and a scan rate of 10 mV/s.

Live cell imaging during the *in situ* stimulation was done by CLSM (LSM900, Zeiss) on a 40 μm z-stack with a pinhole aperture of 1 Airy unit and a time interval of 30 s between two acquisitions with a plan apochromat 20× (NA 0.8) objective. Time-lapse files were processed either with the 3D VTK library of ICY software (image editing) or with ImageJ-FIJI software (image analysis). 3D data sets (z-stacks) were visualized as 2Ds by using the maximum intensity z-projection. 2D data sets were thresholded with a mean function from the background and binarized. Pore outlines were finally displayed, and porosity variations over time were measured.

## Results and Discussion

### Fabrication of 4D Electroactive PolyHIPE/PEDOT Scaffolds

**Figure 1:**
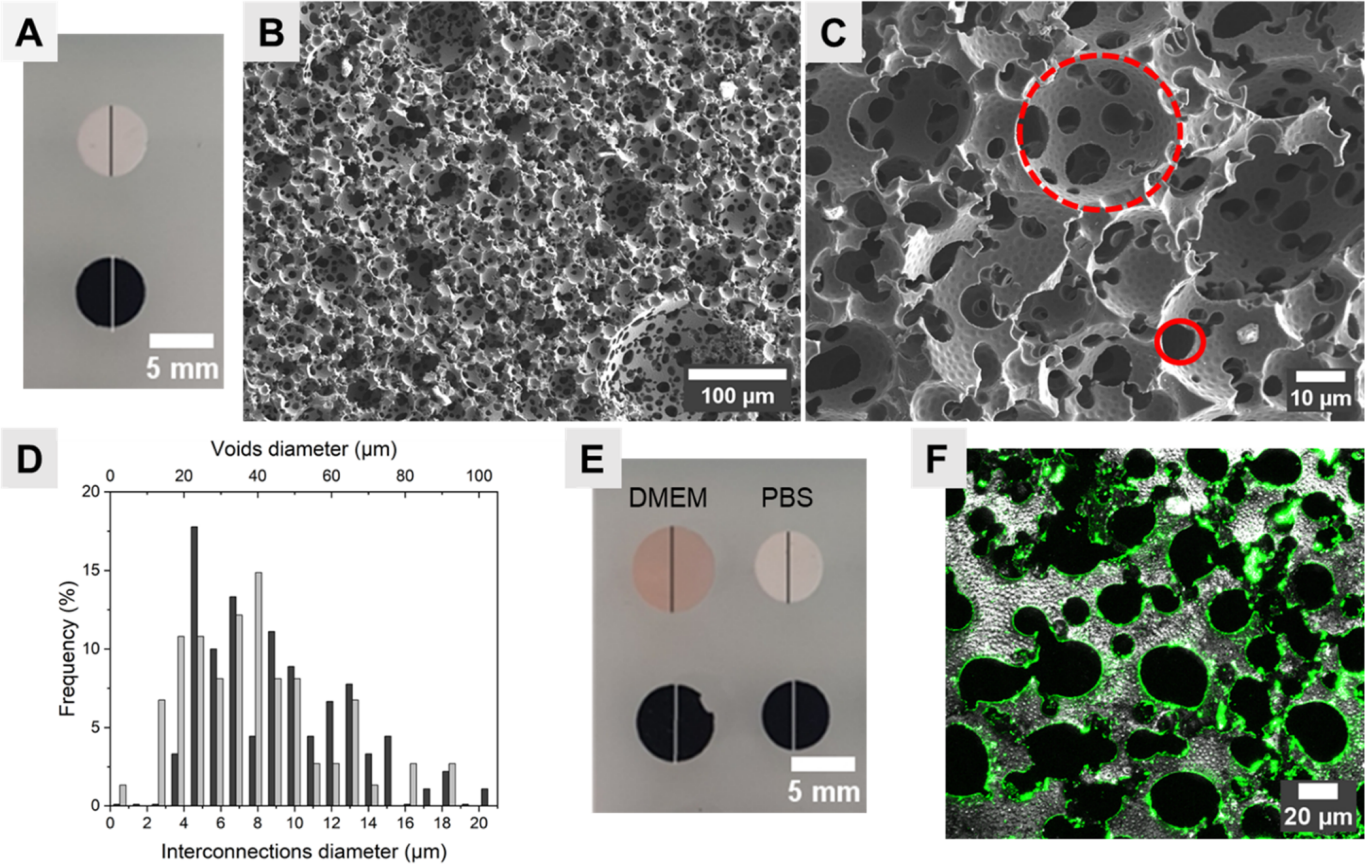
(A) Photograph of polyHIPE polymer 3D scaffolds (white) and polyHIPE/PEDOT scaffolds (black) in dry condition. (B, C) SEM micrographs of polyHIPE/PEDOT scaffold cross sections highlighting the voids (dashed red circle) and interconnections (red circle). (D) Voids (gray) and interconnections (dark gray), diameter analysis. (E) Photograph of polyHIPE and polyHIPE/PEDOT scaffolds immersed in complete cell culture medium (DMEM + 10% FCS) and PBS. (F) Confocal image of the polyHIPE/PEDOT scaffold cross section (gray) functionalized with fluorescent fibronectin (green).

The synthesis of porous polyHIPE scaffolds is based on an emulsion templating method where a continuous external phase (polymer) in a low proportion is emulsified with a high proportion of a dispersed internal phase (water) and then polymerized. As reviewed by Dikici et al., the choice of the continuous phase-composing polymer is a key step to predetermining the properties of the templated scaffolds.^37^ Here, passive polyHIPE scaffolds were successfully produced from PEGDA (acrylate) cross-linked with TMPTMP (thiol) as a white, flexible, and highly stable membrane of 10 cm^2^ with a thickness of 0.5 mm. Such passive 3D polyHIPEs were shown to constitute favorable *in vitro* scaffolds for cell hosting, like stem cells.^14^ In the current study, the polyHIPE 3D surface was further improved using a functionalization with the conductive polymer PEDOT, which provided both a cell-friendly surface and value-added properties consisting of electromechanical responsiveness. PEDOT was incorporated into the polyHIPE scaffolds according to a previously reported procedure.^34^ Typically, the PEDOT incorporation follows a two-step process, starting with the swelling of the polythioether walls of the polyHIPE with vapors of EDOT monomer under static vacuum, followed by the oxidative polymerization of EDOT monomer into PEDOT chains thanks to the exposure of the scaffold to an aqueous iron chloride solution. PEDOT chains are then formed and interpenetrated within the polyHIPE 3D walls, turning them into an electroactive 4D polyHIPE/PEDOT scaffold. PEDOT incorporation colored the membrane black, with a final thickness of 0.7 mm. The functionalized scaffold was easy to handle manually and could be shaped to fit into classical cell culture wells (Figure 1A).

The obtained polyHIPE/PEDOT scaffolds displayed the typical highly porous morphology due to the vacant space released by water droplets of the internal phase.^14,16,37^ Based on the initial internal phase proportion in the HIPE used as a template, the porosity of the polyHIPE was about 80%. Interestingly, the functionalization with PEDOT using vapor-phase swelling/oxidative polymerization allowed a uniform deposition in the whole polyHIPE structure without obstructing the pores (Figure 1B). To avoid any confusion, the same terminology as proposed by Zhang et al.^38^ was adopted for the polyHIPE description, where the term “‘void’” is used to designate the pores generated by the internal phase droplets; the term “interconnection” is used to describe the void connecting holes in the polyHIPE/PEDOT scaffold (Figure 1C). Image analyses show a void size average of about 100 ± 10 μm and a great network of interconnections of 10 ± 1 μm in diameter (Figure 1D). This range of porosity is consistent with typical human cell dimensions, which are around 15–25 μm in diameter, rarely further than 0–50 μm from another cell,^39^ and require pores of 5–250 μm to facilitate cell infiltration.^12^ Young’s modulus of the interpenetrated PEDOT layer functionalizing the polyHIPE walls was estimated to be around 33 MPa thanks to a custom-made protocol (Supporting Information Figure S1).

In order to use polyHIPE/PEDOT scaffolds as *in vitro* cell culture platforms, they should be free of microorganism contamination. Since the impact of the sterilization procedure on the polyHIPE structure remains poorly described among the currently available techniques, polyHIPE/PEDOT scaffolds were sterilized according to a two-step procedure that combines immersion in 70% ethanol as previously done followed by a heat treatment since it is a clinical-grade requirement.^37^ Interestingly, this sterilization process did not alter the original porous structure of both polyHIPE and polyHIPE/PEDOT scaffolds, as shown by SEM analyses (Supporting Information Figure S2).

Both polyHIPE and polyHIPE/PEDOT scaffolds behave as hydrophilic materials, and they slightly swelled about +13 and +7% in diameter, respectively, when immersed in complete cell culture media (DMEM + 10% FCS), which contain a mixture of biomolecules and a great diversity of ions (Figure 1E). The difference in the swelling ratio between polyHIPE and polyHIPE/PEDOT scaffolds can be explained by the slightly higher rigidity of the polyHIPE/PEDOT scaffolds induced by the well-known π-stacking of PEDOT chains.^41^ In both cases, the swelling of polyHIPE and polyHIPE/PEDOT indicates their loading with nutrients and bioactive factors from immersion in the cell culture medium. Besides, when fibronectin, a major glycoprotein of the *in vivo* cell microenvironment, is fluorescently labeled and supplied in solution to polyHIPE/PEDOT scaffolds, a cross-sectional analysis showed that it was adsorbed into polyHIPE/PEDOT and homogeneously coated the whole surface of the holes, as shown in Figure 1F. This biofunctionalization with biomolecules from the cell culture medium could create a nutrient-rich microenvironment for cell hosting and lead to the expectation of a better environment for further cell culture within scaffolds.^14^

### PolyHIPE/PEDOT Scaffolds as Promising Candidates for 3D Cell Culture

**Figure 2:**
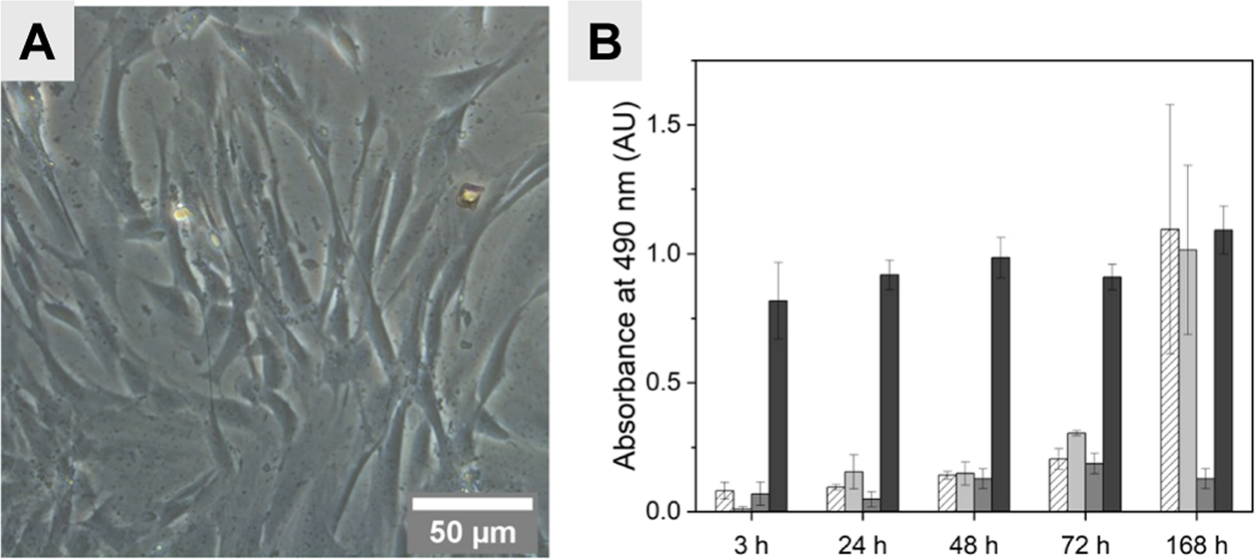
(A) Phase contrast microscopy of BJ cells exposed to preconditioned media of polyHIPE/PEDOT scaffolds during 48 h. (B) LDH activity of BJ cells cultured onto plastic (white dashed), polyHIPE (light gray), and polyHIPE/PEDOT scaffolds (gray) compared to the positive control (dark gray).

The adequacy of polyHIPE/PEDOT scaffolds with the characteristics of a cell culture substrate was first validated by testing the effect of preconditioned cell culture media. When scaffolds were immersed for 24 h in complete cell culture medium, pH, a major physicochemical parameter in cell culture, remained stable around the physiological value. Then, adherent fibroblasts were exposed for 48 h to the preconditioned cell culture medium. The cells remained viable and exhibited a typical mesenchymal morphology, similar to fibroblasts in a classical complete culture medium (Figure 2A).

To evaluate the cytocompatibility of polyHIPE/PEDOT scaffolds, fibroblast cells were cultured onto polyHIPE/PEDOT scaffolds in comparison with polyHIPE alone (nonfunctionalized with PEDOT) and conventional 2D cell culture support (Figure 2B). The LDH activity was analyzed within the cell supernatants to assess cell integrity loss. Up to 72 h, the LDH activity in supernatants from cells cultured on polyHIPE and polyHIPE/PEDOT remained weak and similar to the background signal of the assay measured in the classical 2D culture control. Both were lower than positive LDH release control from dead cells, confirming the polyHIPE and polyHIPE/PEDOT cytocompatibility after 3 days of culture. After 1 week (7 days), the measured LDH activity from polyHIPE or classical 2D culture reached the positive dead cell control value, whereas LDH activity from cells in contact with polyHIPE/PEDOT remained lower. Since the observed cytotoxicity in the classical 2D culture condition could result from a lack of nutrients in the cell culture medium, the lower LDH release from cells cultured on polyHIPE/PEDOT scaffolds compared to that of polyHIPE condition could be related to the conductive property of PEDOT, which could provide physical cues to cells.^33^ This property could increase cell viability over longer culture times compared to “passive” polyHIPE scaffolds and confer better biocompatibility to polyHIPE functionalized with PEDOT.

Then, fibroblasts were seeded onto polyHIPE/PEDOT scaffolds to investigate the capacity of the 3D porous scaffolds structure to support efficient cell colonization. Fibroblasts reside *in vivo* in a 3D interconnected microenvironment. They are mechanosensitive^42,43^ and were previously used to test the biocompatibility of varied 3D porous scaffolds such as thiol–acrylate polymerized polycaprolactone polyHIPEs.^17,40^ 40,000 or 100,000 cells were seeded to investigate cell–scaffold interactions in the range previously used in other studies.^17,40^ We chose to seed fibroblasts at low cell densities to maintain them as disseminated cells, thereby preventing the establishment of cell–cell junctions. *In vivo*, fibroblasts reside mainly as sparse cells within connective tissues.^44^ For instance, Miller et al. estimated the number of fibroblasts in fresh dermis to be around 3,000 cells/mm^3^.^45^ Our seeding conditions align with this density range. For example, in the “Quantitative analysis of the cell penetration”, 100,000 cells were seeded onto scaffolds with an 8 mm diameter and a thickness of 0.7 mm, resulting in a density of 2800 cells/mm^3^. Moreover, an excessive density of fibroblasts (which could promote cell–cell contacts as observed in 2D cultures) may also promote a more fibrosis-related phenotype (α-SMA, contractility, proliferation, etc.).^46^ Furthermore, cells were directly seeded without forming drop-casts or performing a preincubation step to avoid any bias in cell penetration due to a cell concentration effect.

**Figure 3:**
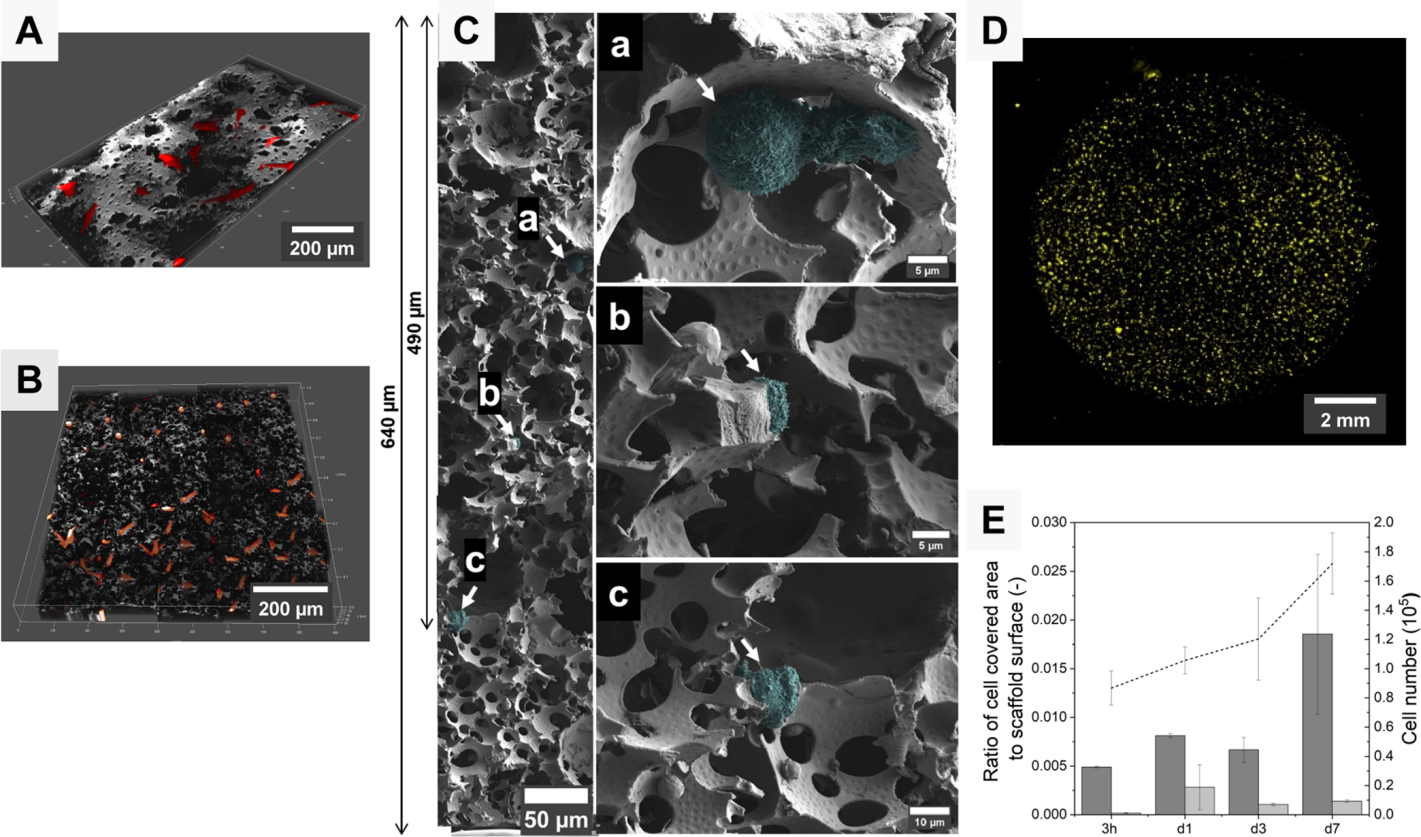
(A, B) 3D confocal images of red TTFLUOR HDF cells seeded and incubated for 24 h on the surface of polyHIPE/PEDOT scaffolds. Surface areas (A) 0.21 mm^2^ and (B) 1 mm^2^ in size. (C) SEM images of three areas with adherent BJ cells in a polyHIPE/PEDOT cross section after 3 h of cell seeding. Images were false-colored for clear visibility of the cells (cyan) on the scaffold surface. (D) Fluorescence wide-field image of the full surface (top) of a polyHIPE/PEDOT scaffold incubated 3 h with red TTFLUOR HDF cells. (E) Ratio of the cell-covered area at the top (dark gray) and the bottom (bright gray) of polyHIPE/PEDOT scaffolds seeded with red TTFLUOR HDF cells (*n* = 2, ± standard deviation) compared to the total number of cells quantified by DNA analysis (dashed line, *n* = 3, ± standard deviation).

As shown on 3D representations of confocal optical sections (100 μm depth) from low (Figure 3A) or high (Figure 3B) cell number fields, numerous fibroblasts can be found in the first layer of the polyHIPE/PEDOT scaffolds 24 h after cell seeding. Fibroblasts displayed an extended, typical morphology of attached and spread cells. It illustrates a rapid and efficient fibroblast cell interaction with the polyHIPE/PEDOT surface. On the other hand, a SEM cross-sectional analysis highlighted the early start of an in-depth cell migration process since cells were found up to 490 μm from the top of the material 3 h after cell seeding (Figure 3C). In the depth of the scaffolds, cells were found within voids, where they exhibited a nuclear round zone (Figure 3C(a)) and a membrane flat morphology in close vicinity with the voids’ walls (Figure 3C(a–c)). This shape is typical of the firm attachment and early spreading of cells. The presence of fibroblasts within scaffolds at a longer time was confirmed by fluorescence microscopy analysis of cross section (Supporting Information Figure S3).

The whole scaffold colonization was then analyzed over 7 days by using quantitative imaging. For this, red TTFLUOR HDF cells were seeded on 8 mm diameter polyHIPE/PEDOT scaffolds (Figure 3D). For the cell migration through the porous structure, the full top surface, where the cells were seeded, and the bottom surface, with few to no transmigrated cells at t_0_, were acquired with a wide-field microscope. At the same time, the total number of cells was determined using a DNA assay (Figure 3E). After 3 h, DNA quantification indicated that more than 85,000 cells out of the 100,000 seeded cells were present within the whole polyHIPE/PEDOT scaffold. This observation that around 85% of the cells adhere after 3 h could be explained by the washing of polyHIPE/scaffold prior to analysis, which induced a classical loss of nonadhesive cells at an earlier time after cell seeding. From day 1 to day 7, the number of total cells increased from 110,000 cells to 170,000 cells (corresponding to one doubling time from the attached cells after cell seeding). These results show moderate cell proliferation, even though cells were sparse within the scaffold. At the same time, the cell-covered area on the top of the polyHIPE/PEDOT scaffold slightly increased from 3 h to day 1 after cell seeding, indicating that cell adhesion was completed on day 1 since the total cell number corresponded to the number of seeded cells. On day 3, the decrease of the cell-covered area at the top of the scaffold could be a sign of cell migration due to the penetration of cells inside the scaffold since the total number of cells increased slightly on day 3 according to DNA analysis. Furthermore, the increase in the cell-covered area and the total cell number at day 7 can be traced to cell spreading and proliferation. The cell-covered area at the bottom of the scaffolds remained small over time. These data suggested that the seeded fibroblasts were able to attach and infiltrate the polyHIPE/PEDOT scaffolds, where they could grow both at the surface and inside the scaffolds, while only a few cells fully transmigrated at the bottom face scaffolds.

Interestingly, when colonized cells were subjected to the action of trypsin, cleaving the cell–substratum interaction, they became round but remained trapped within the scaffold (Supporting Information Figure S4). This could confirm that the displacement of cells toward the polyHIPE/PEDOT scaffold during infiltration is an active process that necessitates cell deformation and cell/substratum interaction rather than a passive colonization resulting from cell flushing (Supporting Information Figure S5). Even if these results remain to be fully understood, they provide some key elements about the kinetic and penetration processes in 3D porous scaffolds that are poorly described in the literature.

On the other hand, it highlights that the open porosity of the developed 3D polyHIPE/PEDOT allowed efficient cell penetration and viability in depth (490 μm after 3 h). The depth of penetration of fibroblasts is comparable to those described in the literature but they migrate faster. For example, preosteoblasts reached a depth of 200 μm after 1 week and 450 μm after 14 days^47^ while human dermal fibroblasts were found up to 250 μm after 7 days within polycaprolactone-based polyHIPE.^15^

### PolyHIPE/PEDOT Scaffolds Support 3D Cell Spreading

**Figure 4:**
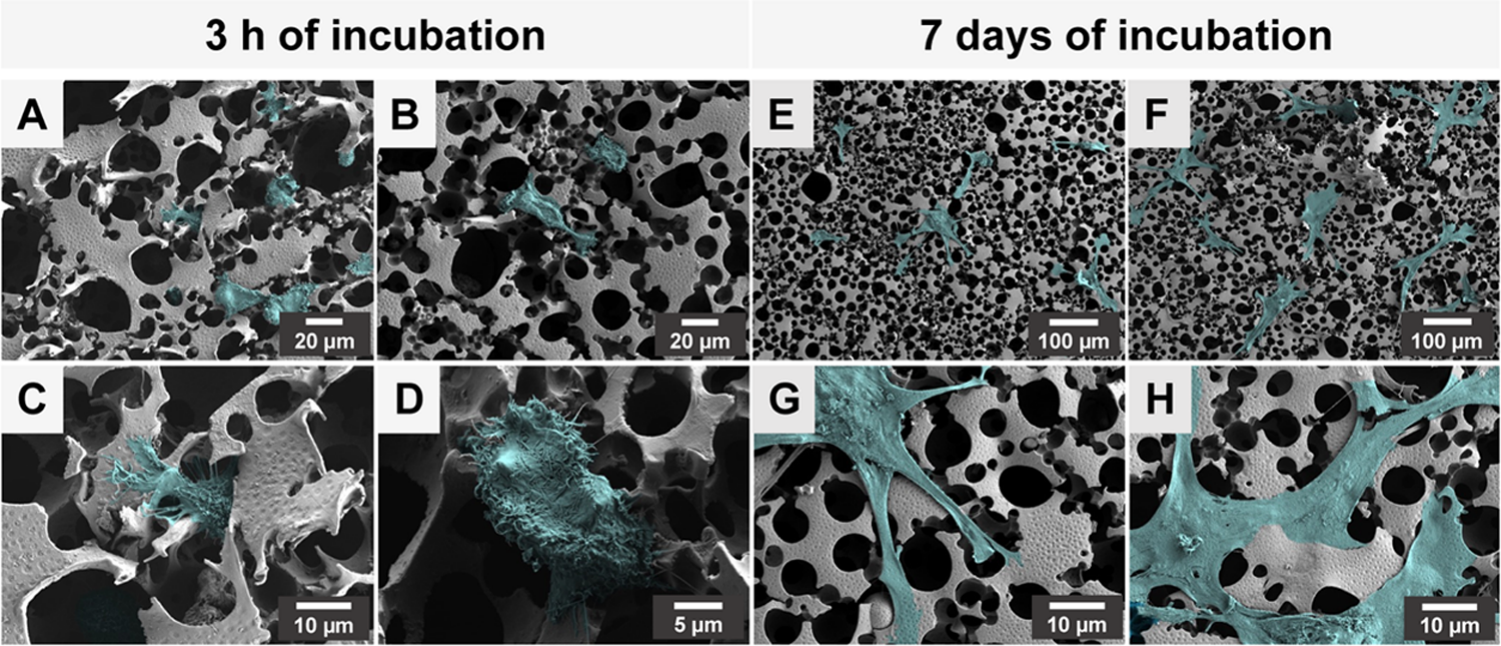
SEM images of BJ cells seeded on polyHIPE/PEDOT scaffolds after 3 h (A–D) and 7 days (E–H) of incubation. Images were false-colored for clear visibility of the cells (cyan) on the scaffold surface.

**Figure 5:**
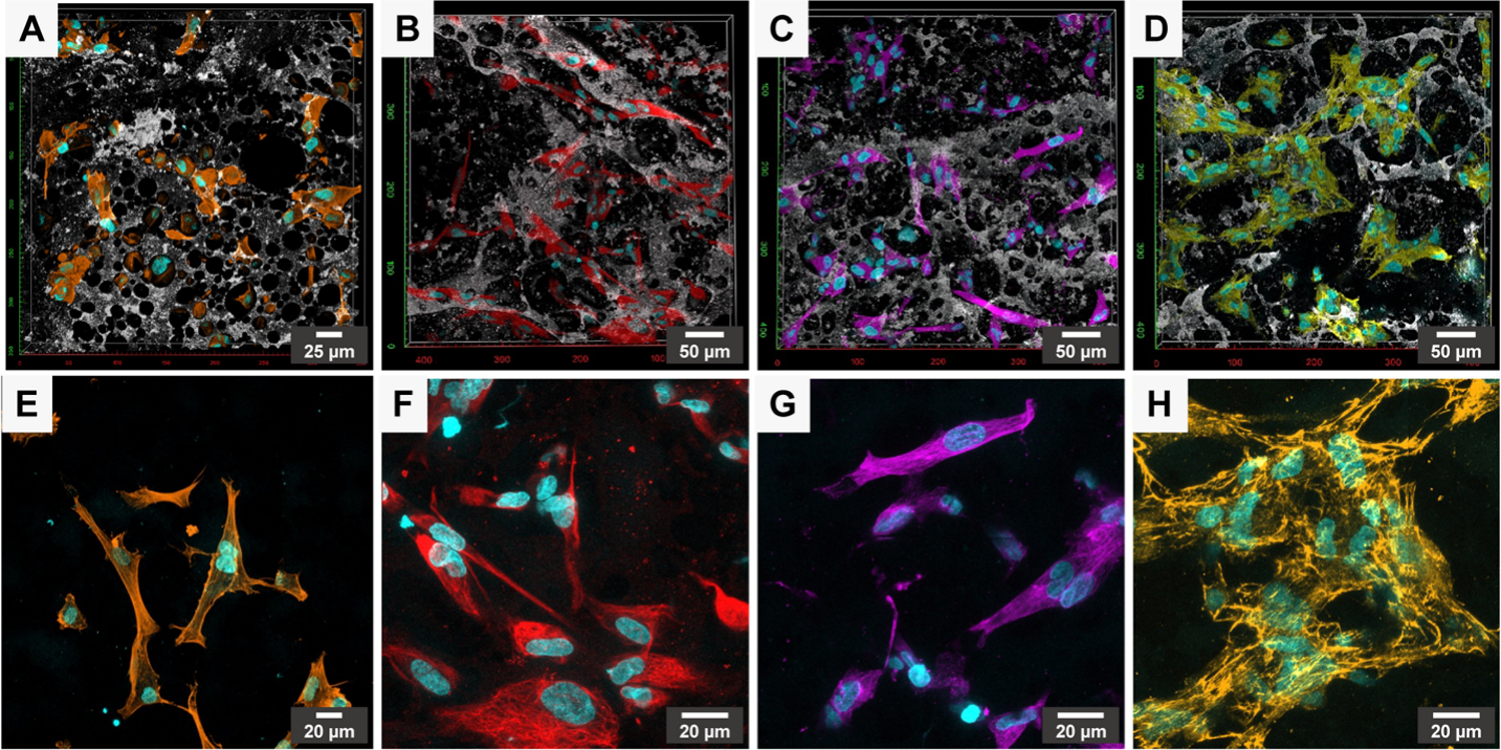
(A–D) 3D visualization of confocal images of BJ cells spread on the surface of polyHIPE/PEDOT scaffolds after 24 h of incubation. (E–H) Corresponding 2D visualization of the confocal images by creating the maximum intensity z-projection for each fluorescence channel without topography. DNA (cyan), F-actin (orange), vimentin (red), tubulin (magenta), cellular fibronectin (yellow), and topography (gray). Surface areas (A) 0.1225 mm^2^ and (B–D) 0.36 mm^2^ in size.

*In vivo*, fibroblasts are fully surrounded by a 3D matrix.^9^ Within this 3D environment, fibroblasts exhibit a morphology that differs from the 2D one.^10^ As the cell shape could, in turn, impact cellular activities,^48^ cell morphologies while interacting with polyHIPE/PEDOT scaffolds were observed by SEM (Figure 4) and CLSM (Figure 5).

3 h after cell seeding, fibroblasts began to spread (Figure 4A–D). In contrast to confinement-constrained morphologies, cells displayed a round, elongated corpus and exhibited numerous thin plasma membrane extensions. These active protrusions, which are characteristic of membrane dynamics during active cell crawling,^49^ were observed all around the cell periphery. Among membrane protrusions, some of them extended toward the voids of the scaffold, while others established thin contacts with the scaffold surface. After 7 days of culture (Figure 4E–H), extensive cell spreading was observed and cells homogeneously covered the surface of the polyHIPE/PEDOT scaffold. While spreading on the outer surface of the scaffold, cells adopted a more spindle-shaped and thin-flattened morphology with large lamellar regions and the disappearance of the nuclear round-shaped zone. Elongated membrane protrusions radiated into interconnections and were anchored to the side walls. In some areas, the interface between the plasma membrane and the scaffold was undistinguishable, highlighting a firm adhesion essential for their function.^8,49^

The cell cytoskeleton is an important machinery for sensing the cell microenvironment and establishing the cell shape, modulated by its resistance to deformation. Cell’s shape in turn is intimately linked to cell’s behavior. The cytoskeleton is composed of three types of cytoskeletal proteins’ polymers: (i) filamentous actin (F-actin), which shapes the cells and allows spreading and migration; (ii) microtubules, which display a key role in cell division and vesicular trafficking, in particular, to deliver adhesive molecules to the plasma membrane; and (iii) intermediate filaments that allow cell resistance.^43,50^ All of the cytoskeletal components are involved in the mechanosensitivity of fibroblasts.^51−53^ So, the molecular organization of cytoskeleton components during fibroblast cell interactions with polyHIPE/PEDOT scaffolds was explored. For that, the fluorescent labeling of F-actin, tubulin, and vimentin as respective components of actin filaments, microtubules, and intermediate filaments (specifically from mesenchymal cells like fibroblasts) was performed 24 h after cell seeding and analyzed by confocal microscopy. Nuclei were counterstained with DAPI (Figure 5).

At low magnification, whatever the labeled component (Figure 5, top panel), several cells colonized the scaffold in the focal plane (scaffold surface) or out-of-focus planes (deeper within the scaffold). Within the 3D scaffold, fibroblasts adopted a more bipolar or stellate spindle-shaped morphology than on the surface. This morphology is evocative of embedded fibroblasts in 3D matrices and was described to be close to cell morphologies encountered *in vivo*.^10,43,48^ At higher magnification (Figure 5, lower panel), actin filaments were concentrated at the cell periphery in the cortical regions underlying the plasma membrane. Within cells’ cytoplasm, actin bundles were thin, and only a few stress fibers could be identified (Figure 5E). Labeled microtubules (Figure 5G) were organized along a network that radiates from the nucleus toward the plasma membrane, while intermediate filaments of vimentin formed an extensive network within the whole cell body (Figure 5F). Such location and organization of cytoskeletal components are related to the common 3D morphology of fibroblasts. Thus, polyHIPE/PEDOT scaffolds provided both space for spreading and sufficient interaction inputs to allow ventral and dorsal anchorage of fibroblasts, resulting in their 3D morphology even if cells are sparse. It will allow fibroblasts to sense and respond to spatial inputs from polyHIPE/PEDOT scaffolds.^48^ As cell shape is intimately linked to cell activity, a staining of fibronectin as a marker of fibroblast functionality (which secretes and organizes it) was performed (Figure 5D–H). Within polyHIPE/PEDOT scaffolds, a dense and fibrillar fibronectin network surrounding cells and numerous fibronectin fibrils associated with cell extensions were observed (Figure 5H). Thus, fibroblasts could assemble fibronectin and incorporate it in a cell-derived extracellular matrix, keeping one of the key roles they have *in vivo*.^54^

Fibroblast behavior within polyHIPE/PEDOT scaffolds (attachment, penetration, morphology, and matrix organization) complies with the requirements in cell culture applications as passive 3D porous and interconnected scaffolds. Reciprocally, a better understanding of microenvironmental mechanical cues that can trigger fibroblast-specific behavior and their activation (*e.g*., to become myofibroblasts often associated with excessive proliferation) would have a significant impact on pathologies such as fibrosis or reactive cancer-associated stroma. In this case, the development of tunable and responsive systems to control and tailor porosity size and mechanical properties will be valuable.^55^ Therefore, the electromechanical responsiveness of polyHIPE/PEDOT, especially the integration of dynamics in cell cultures, could be of great interest for the evaluation of such studies.

### Electrochemical Stimulation and Electromechanical Response of PolyHIPE/PEDOT Scaffolds

**Figure 6:**
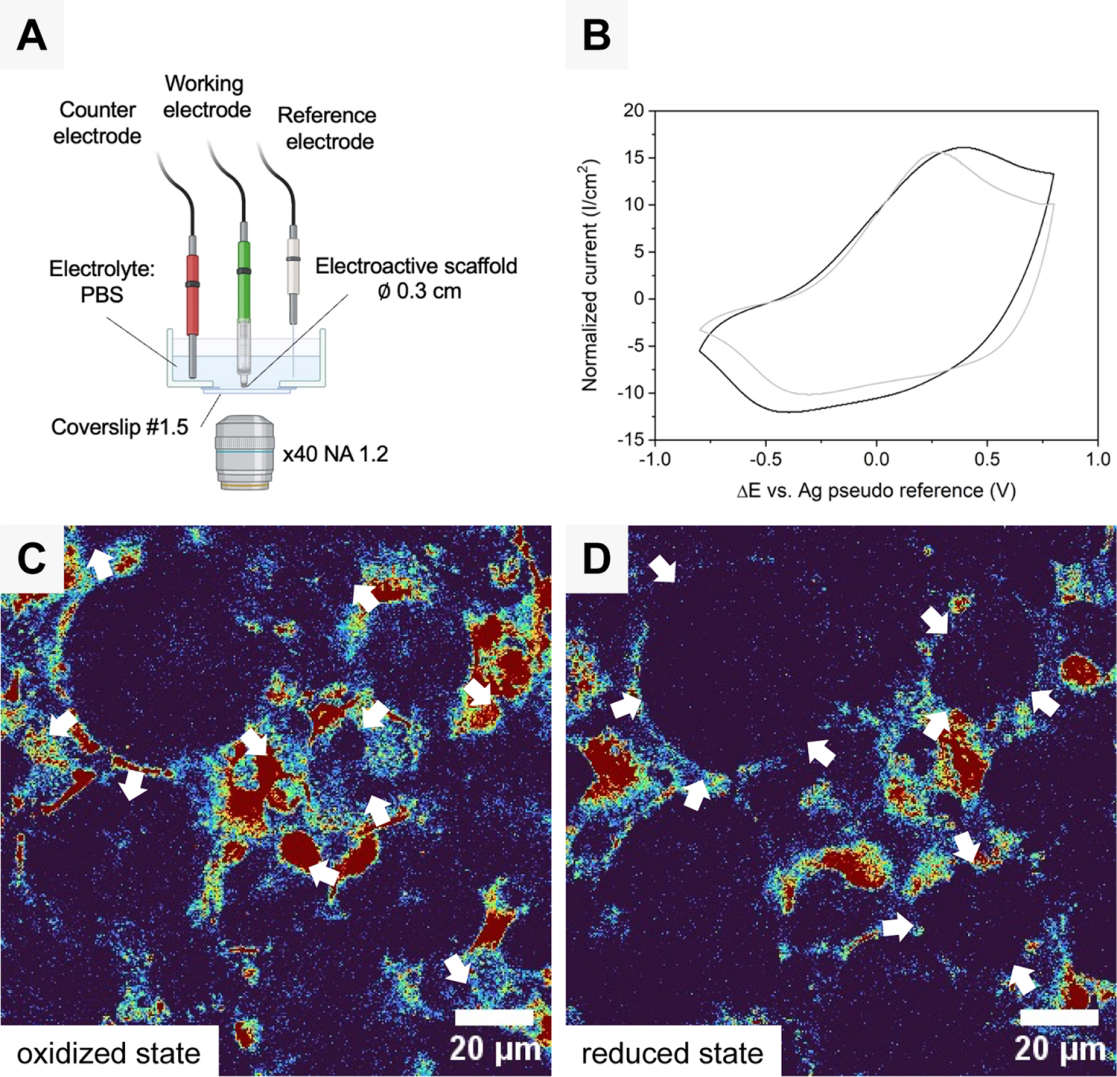
(A) Experimental three-electrode setup for monitoring the mechanical response of the polyHIPE/PEDOT scaffold. (B) PolyHIPE/PEDOT scaffold electroactivities analyzed by CV in PBS (black line) and complete cell culture medium (gray line) for a three-electrode setup at a scan rate of 10 mV/s and a potential window of 0.8 V/–0.8 V (Δ*Evs* Ag pseudoreference = 1.6 V). (C, D) Confocal topography images of oxidized (expanded) at 0.8 V and reduced (compressed) at −0.4 V states of polyHIPE/PEDOT scaffold in complete cell culture medium by alternating the potential each 60 s for a duration of 3 cycles. Images were false-colored by a “rainbow“ lookup table to better emphasize the intensity of low intensity (blue colors) and high intensity (red colors). White arrows show the deformation of the scaffold during actuation.

First, the electroactivity of polyHIPE/PEDOT scaffolds (i.e., the ability of the scaffolds to be electrochemically oxidized or reduced) was evaluated by cyclic voltammetry (CV) in a classical three-electrode setup (Figure 6A) in PBS or in complete cell culture medium, which contains a great variety of macromolecules and ions. FCS, supplemented as a serum component in the complete cell culture medium, contains many proteins, including adhesive proteins like albumin and fibronectin, which can be adsorbed on the scaffold surface and could influence the electroactivity. Whatever the conditions, the voltammograms displayed typical oxidation and reduction peaks associated with PEDOT.^56^ More defined oxidation and reduction peaks are observed for the polyHIPE/PEDOT scaffold in complete cell culture medium, indicating a higher electroactive response of the material than in PBS. This finding can be explained by a more diverse ion concentration in the complete cell culture medium, which facilitates the ion-exchange process occurring during the redox stimulation and subsequently increases the current density. Accordingly, the electroactivity was not reduced by the possible absorption of proteins such as albumin and fibronectin. In summary, CV showed that the polyHIPE/PEDOT scaffold is electroactive and accessible to ion insertion/expulsion in a complete cell culture medium.

The electromechanical response, which takes the form of a volumetric variation of the scaffold, was observed by CLSM in reflection mode (Figure 6C,D). By alternating the potential applied to the scaffold at the working electrode (WE) from 0.8 to −0.4 V for 60 s per oxidation/reduction step, both oxidized and reduced states were observed alternatively. The bright red and yellow/green areas correspond to the polymer scaffold, and the dark blue areas correspond to the voids and interconnections in the polyHIPE structure. When neutral PEDOT^0^ is oxidized to PEDOT^+^ under positive voltage, the polyHIPE/PEDOT scaffold expands due to the anion insertion mechanism, ensuring electroneutrality. The oxidation process induces a volume increase in the matrix and an increase of porosity. When the oxidized PEDOT^+^ is reduced toward neutral PEDOT^0^ by applying a negative voltage, the porous scaffold contracts because anions are being expelled and smaller pore sizes can be observed. The motion due to contraction/expansion of the PEDOT happened throughout the whole sample. It was reversible and easily trackable with a classic confocal microscope without the need for fast acquisition devices like a galvanometric stage or a resonant scanner (Supporting Information Movie S1).

### PolyHIPE/PEDOT Scaffold Allows Us to Monitor Cell Stimulation *In Situ* and In Real Time

**Figure 7:**
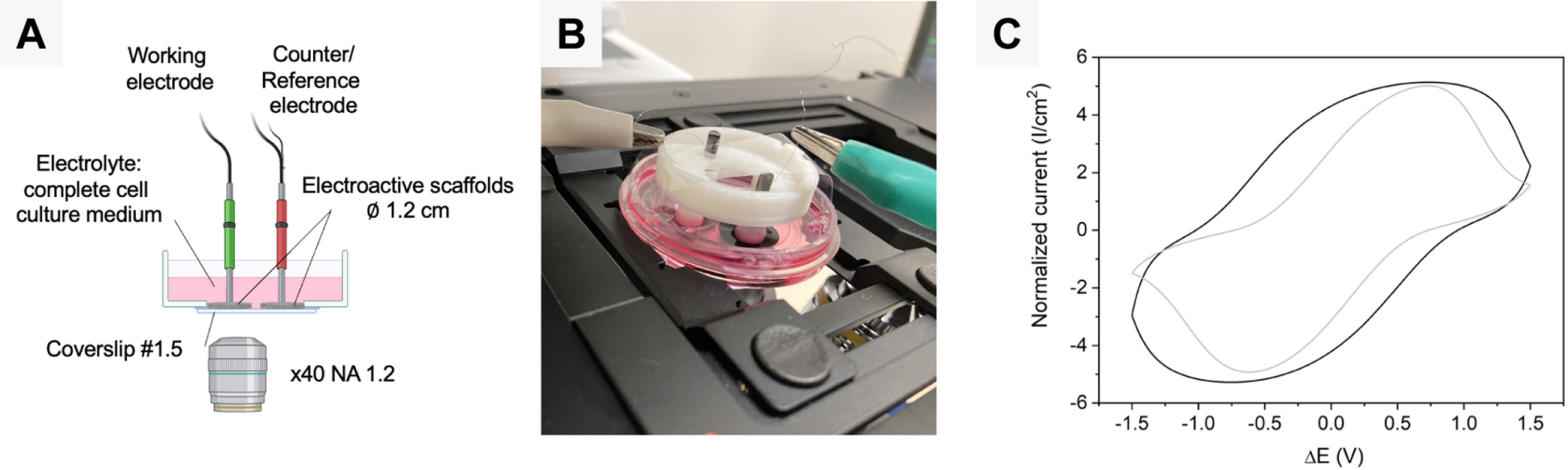
(A) Experimental two-electrode setup for monitoring the mechanical response of the polyHIPE/PEDOT scaffold and the dynamics of seeded cells. (B) Photograph of the two-electrode setup with the 3D-printed holder ready for image acquisition onto the stage of a laser scanning confocal microscope. (C) PolyHIPE/PEDOT electroactivities were analyzed by CV in PBS (black line) and complete cell culture medium (gray line) for a two-electrode setup at a scan rate of 10 mV/s and Δ*E* = ± 1.5 V.

The electromechanical response of polyHIPE/PEDOT scaffolds and monitoring of associated cell deformations were observed by CLSM using a customized setup in a two-electrode configuration. This setup was composed of two glassy graphite rods used as current collectors in contact with two large, similar in the specific surface area and porous scaffolds (diameter = 1.2 cm from the same batch of synthesis), playing, respectively, the role of working and counter electrodes (Figure 7A). To ensure an efficient connection of both electrodes in the glass-bottomed Petri dish, they were inserted in a 3D-printed holder (Figure 7B). Smart Again filament was chosen for 3D printing because of its high stability and printability. Its cytocompatibility was checked with LDH and DNA quantifications, where both LDH activity and the total number of cells were in the same range as in the control cell culture (Supporting Information Figure S6). This setup allowed a charge balance during redox reactions occurring simultaneously at both electrodes, which are endowed with close to identical specific surfaces (around 200–700 m^2^/g).^57,58^ The observed contraction/expansion of the scaffold at the WE produced by electrostimulation is *de facto* not limited by the current crossing the counter electrode (CE). Thus, this customized improvement of the previous setup allowed for the stimulation of larger scaffolds and consequently higher cell densities, which are necessary for *in vitro* studies.

The operation of the customized setup in a two-electrode configuration was analyzed by using CV. Symmetric voltammograms for polyHIPE/PEDOT scaffolds in PBS and complete cell culture medium display the characteristic behavior of electroactive materials in a two-electrode configuration (Figure 7C). As in the previous setup, polyHIPE/PEDOT scaffolds showed in complete cell culture medium enhanced and more defined oxidation and reduction peaks, indicating a higher electroactive response of the material than in PBS.

**Figure 8:**
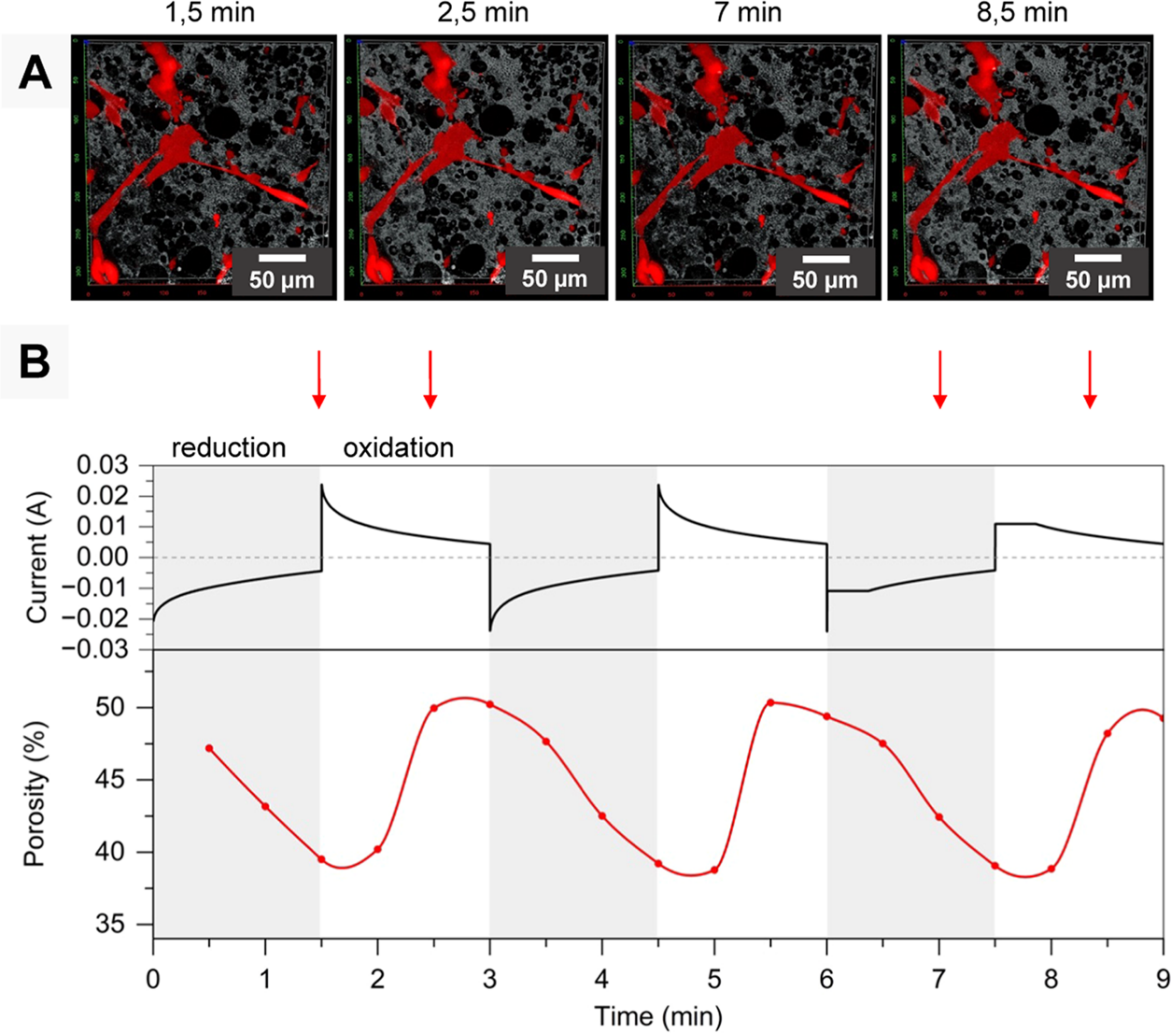
(A) *In situ* mechanical stimulations of red TTFLUOR HDF cells seeded on polyHIPE/PEDOT scaffolds stimulated for 9 min observed directly during stimulation under CLSM. 3D visualization of confocal images with a surface size of 0.09 mm^2^. (B) The electrical signal of stimulation (alternating the potential for 3 cycles, 90 s per oxidation/reduction step at Δ*E* = ± 1.5 V; black line) and the corresponding variations of porosity of the polyHIPE/PEDOT scaffold due to the electrical stimulation (per oxidation/reduction step, 3 z-stacks were acquired with a duration of 30 s, red line). Gray areas highlight reduction steps (expansion), and white areas highlight oxidation steps (contraction). Red arrows indicate the images’ time points 1.5, 2.5, 7, and 8.5 min.

Red TTFLUOR HDF cells were seeded onto polyHIPE/PEDOT scaffolds and allowed to adhere and spread for 3 h prior to *in situ* electromechanical stimulation. Cell-seeded scaffolds were electrically stimulated by alternating the potential for 3 cycles (Δ*E* = ± 1.5 V) for 9 min to monitor both mechanical response and cell behavior in real time (Figure 8A and Supporting Information Movie S2). The potential difference of ±1.5 V fits in the range of published stimulation (i.e., electrical fields of strengths as large as 2 V/cm were reported at wound sites where fibroblasts can be identified).^59^ We observed no difference between the actuation of the bare or seeded with cells polyHIPE/PEDOT scaffolds. This is consistent with the observation of del Valle et al., who showed that PEDOT electroactivity was not modified by covering with cells or proteins.^60^ This observation is extended here to the actuation behavior. Volume variation and porosity change during actuation remained in the same range of 10% of polyHIPE/PEDOT as previously measured.^34^

Electrical stimulation caused the contraction of the scaffold in the reduced state (1.5 and 7 min) or its expansion in the oxidized state (2.5 and 8.5 min), resulting in electromechanical stimulation of the cells (Figure 8A). During stimulation, the cells remained on the surface of the scaffold and there was no evidence of cell detachment. The actuation response (change in porosity) of the polyHIPE/PEDOT scaffold followed electric stimuli (Figure 8B), meaning, while the scaffold was reduced by the electrical stimuli (Figure 8B, gray area), a decrease in porosity was consequently observed (Figure 8B, red line in the gray area), following the actuation hypothesis that reduction leads to compression of the scaffold. Similarly, while the scaffold was oxidized by electrical stimuli (Figure 8B, white area), an increase in porosity could be detected (Figure 8B, red line in the white area), indicating that the scaffold was expanded. Such stimulation conditions displayed no cytotoxicity for fibroblasts since LDH activity remained similar to the control immediately or 3 h after stimulation (Supporting Information Figure S7).

Even though it remains to be further investigated, our described stimulation setup led to the cells experiencing an approximate electrical field of 2 V/cm. Such an electrical field can influence the cell behavior like cell adhesion, morphology, or migration, along with cytoskeleton modification. Finkelstein et al. investigated the electrical field-mediated motility (galvanotaxis) of 3T3 fibroblasts in sparse cells and wounded monolayers. They found that cells migrate faster and toward the electrical field and demonstrated the possibility of using the electrical field to engineer wound healing constructs.^61^ Titushkin et al. evaluated the effect of the electrical field on the actin cytoskeleton. By applying an electrical field of up to 2 V/cm to human mesenchymal stem cells (hMSCs) and osteoblast, they could observe an increase in the intracellular Ca^2+^ levels, which led to depolymerization of the F-actin followed by a decrease of cell elasticity. This effect was reversible after 60 min of electrical stimulation. Additionally, the influence of the electrical field led to a loss in membrane tension due to the separation of the cell membrane from the cytoskeleton.^59^

As cells experienced about 10% mechanical deformation through the scaffold during contraction and expansion cycles, the deformation of the polyHIPE/PEDOT scaffold and the induced cell response are in the same range of 2.5–24% deformation as in various studies describing the application of external tensile and compressive forces or shear stress.^1,62^ Compared to other dynamic systems such as deformable substrates,^63,64^ electronic patches,^65^ or commercial devices such as Flexcell^66^ or MechanoCulture,^67^ which permit uniaxial deformation, the motion of the polyHIPE/PEDOT scaffold is multiaxial. It confers on the 3D porous polyHIPE/PEDOT scaffolds a full 4D dynamic and reversible microenvironment for cell culture. While further experiments are needed, for example, preliminary data suggest that cell secretion could be assessed in conditioned media of stimulated cells (Supporting Information Figures S8 and S9), these results provide proof of concept and demonstrate the interest of this smart stimulation device as an *in vitro* cell culture platform. It could enhance and expand several approaches, such as large field of view monitoring (Figure 8A) suitable for drug screening under tensile forces^68^ or single cell imaging (Figure 8B) more dedicated to mechanobiology studies.^64^

## Conclusions

A 4D polyHIPE/PEDOT scaffold was developed and evaluated as a new dynamic cell culture platform. The morphology of the synthesized polyHIPE presents interconnected voids that are compatible with cell infiltration. The electromechanical property was unchanged when scaffolds were immersed in complete cell culture medium. PolyHIPE/PEDOT scaffolds comply with cell culture requirements: they are cytocompatible and enable fast and in-depth cell penetration. Fibroblasts rapidly colonize, adhere, spread, and exhibit typical 3D morphology. PolyHIPE/PEDOT scaffolds present a mechanical response, resulting in pore size variations under low electrical voltage. Stimulation setups were designed and implemented for a confocal microscope. Our devices enabled *in situ* and real-time monitoring of cell dynamics under 3D expansion and compression induced by electromechanical stimulation. Based on our first results, the proposed platform could be used as a tunable tool for mimicking the dynamics of the cell microenvironment for guiding specific cell functions or behaviors during cell differentiation. This 4D polyHIPE–PEDOT-based scaffold paves the way for a broad range of applications such as mechanobiology studies or biomimetic drug screening analysis.

## Supporting Information Available

Determination of Young’s modulus of the PEDOT layer interpenetrated within the PTE host matrix; SEM images of polyHIPE/PEDOT before and after the sterilization process via autoclave; fluorescence microscopy images of surface and cross section of polyHIPE/PEDOT seeded with red TTFLUOR HDF cells; 3D representation of polyHIPE/PEDOT scaffold seeded with BJ cells 45 min after the trypsinization step after 3 h of incubation acquired by confocal microscopy with stained cell nuclei (DAPI); DNA quantification of red TTFLUOR HDF cells seeded on polyHIPE/PEDOT scaffold 24 h after flushing and removing cell suspension or in classical seeding condition; LDH activity and DNA content of 3D printing material (Smart Again); LDH assays before stimulation and 0 or 3 h after a 10 min stimulation; ELISA assay of secreted fibronectin from cells seeded on polyHIPE/PEDOT scaffold without any stimulation and after 5 h of stimulation; and cytokine array of cytokines released by BJ cells on polyHIPE/PEDOT scaffolds stimulated during 5 h or not stimulated (PDF)
Actuation response of polyHIPE/PEDOT scaffold in oxidized and reduced states; confocal microscopy (Movie S1) (AVI)
Actuation response of polyHIPE/PEDOT scaffold seeded with red TTFLUOR HDF cells; confocal microscopy (Movie S2) (AVI)

## Supplementary Material

am3c16686_si_001.pdf

am3c16686_si_002.avi

am3c16686_si_003.avi

## Abbreviations

Δ*E*: potential difference
2D: two-dimensional
3D: three-dimensional
4D: four-dimensional
Ag: silver
CE: counter electrode
CLSM: confocal laser scanning microscope
CV: cyclic voltammetry
DMEM: Dulbecco’s modified Eagle’s Medium
DNA: deoxyribonucleic acid
ECPs: electronic conducting polymers
EDOT: 3,4-ethylenedioxythiophene
EDTA: ethylenediaminetetraacetic acid
FCS: fetal calf serum
HDF: human dermal fibroblast
LDH: lactate dehydrogenase
PBS: phosphate buffer saline
PEDOT: poly(3,4-ethylenedioxythiophene)
PEGDA: poly(ethylene glycol) diacrylate
polyHIPE: polymerized high internal phase emulsion
ref: reference electrode
SEM: scanning electron microscopy
TMPTMP: trimethylolpropane tris(3-mercaptopropianate)
UV: ultraviolet
WE: working electrode
