# Supplementary material for: Electroactive 4D Porous Scaffold Based on Conducting Polymer as a Responsive and Dynamic In Vitro Cell Culture Platform
Source: ACS Appl Mater Interfaces. 2024 Jan 26;16(5):5613–26. doi: 10.1021/acsami.3c16686 (PMC10859895; doi:10.1021/acsami.3c16686)
Supplement: Supplementary file 1 — am3c16686_si_001.pdf [file am3c16686_si_001.pdf]

Supporting Information

Electroactive 4D porous scaffold based on conducting polymer as a responsive and dynamic *in vitro* cell culture platform

*Franziska Hahn [a,b †], Ana Ferrandez-Montero [a,b,c *†], Mélodie Queri [a,b], Cédric Vancaeyzeele [b], Cédric Plesse [b], Rémy Agniel [a *†], Johanne Leroy-Dudal [a †].*

[a] Equipe de Recherche sur les Relations Matrice Extracellulaire-Cellules (ERRMECe), Groupe Matrice Extracellulaire et Physiopathologie (MECuP) , I-Mat, CY Cergy Paris Université, 95000, Neuville sur Oise, France

[b] Laboratoire de Physicochimie des Polymères et des Interfaces (LPPI), I-Mat, CY Cergy Paris Université, 95000, Neuville sur Oise, France.

[c] Instituto de Ceramica y Vidrio (ICV), CSIC, Campus Cantoblanco, Kelsen 5. 28049, Madrid, Spain

* corresponding author

Contact: aferrandez@icv.csis.es ; remy.agniel@cyu.fr

[†] These authors contributed equally to this work

Franziska Hahn and Ana Ferrandez-Montero contributed equally to this paper.

Rémy Agniel and Johanne Leroy-Dudal contributed equally to this paper.

Determination of the Young's modulus of the PEDOT layer interpenetrated within the PTE host matrix.

Measuring the Young's modulus of the PEDOT-PTE walls of the polyHIPE-PEDOT is very challenging. To answer this question, we synthesized PEDOT-PTE non-porous materials from (i) PTE non-porous films with the same chemical composition than the polyHIPE walls and (ii) functionalized the PTE non-porous surface with PEDOT following the same procedure than for the polyHIPE-PEDOT (i.e. EDOT swelling ratio vs PTE film of 120 wt%, oxidative polymerization by immersion in $FeCl_3$ 1.5 M aqueous solution at 40 °C for 3 h). By doing so, the interface between the EDOT-swollen PTE host matrix and the oxidant solution can be reproduced and the resulting PTE-PEDOT non-porous films can then mimic the physicochemical properties of the polyHIPE-PEDOT wall (Figure S1a). By using this chemical synthesis pathway and after cutting the edges of the resulting material, a trilayer architecture is obtained with two PEDOT layers interpenetrated within the PTE host matrix separated by a central layer made of PTE network. The thickness of the interpenetrated PEDOT layer can be estimated by a sulfur mapping of the trilayer cross-section, performed by EDX spectroscopy (Figure S1b). The thickness of interpenetrated PEDOT layers is estimated to be 43 ± 5 µm within the PTE film. The trilayer and the single PTE networks are then characterized by tensile tests in order to estimate the Young's modulus of the PEDOT layer interpenetrated within the PTE host matrix (Figure S1c) by using an additivity model of multilayer materials (equation 1):

$$(1)\quad E_{trilayer} \cdot h_{trilayer} = E_{PEDOT1} \cdot h_{PEDOT1} + E_{PTE} \cdot h_{PTE} + E_{PEDOT2} \cdot h_{PEDOT2}$$

With E_i and h_i being respectively the Young's modulus and the thickness of each layer, i corresponding to the trilayer, the PEDOT layer and the PTE film.

Knowing the Young's modulus of the PTE film and of the trilayer as well as the thickness of each layer, the Young's modulus of the interpenetrated PEDOT layer can be calculated according to the equation 2:

$$(2)\quad E_{PEDOT} = \frac{E_{trilayer} \cdot (2h_{PEDOT} + h_{PTE}) - E_{PTE} \cdot h_{PTE}}{2h_{PEDOT}}$$

The Young's modulus of the interpenetrated PEDOT layer was estimated to be around 33 MPa.

This value corresponds to the mechanical properties of the interpenetrated PEDOT layer after synthesis, usually associated to an intermediate redox state. These measurements have not been performed on fully oxidized or fully reduced PEDOT layers but previous studies on the influence of the redox level on the Young's Modulus of PEDOT layers have shown a moderate variation of the mechanical properties when switching from totally oxidized to the totally reduced state, in the range of 9-23% (Rohtlaid et al., 2021 https://doi.org/10.1002/admt.202001063). By analogy, we can confidently consider that the Young's modulus of the PEDOT layer does not change by orders of magnitude during the electrochemical stimulation (i.e. from stiff to soft). Consequently, the estimated value of the PEDOT Young's modulus (33 MPa) corresponds to the mean mechanical properties perceived by the cells within the polyHIPE-PEDOT walls."

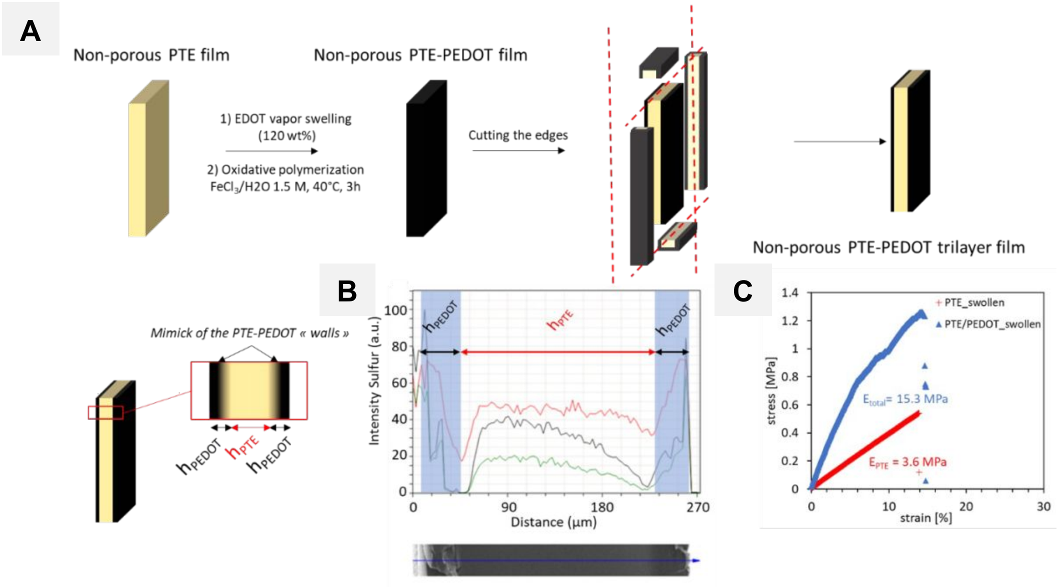

Figure S1. (A) Elaboration pathway of non-porous PTE-PEDOT films mimicking the physicochemical properties of the polyHIPE-PEDOT walls. (B) Energy dispersion spectroscopy of the cross-section of the PTE-PEDOT film with sulfur mapping, allowing to measure the thickness of the interpenetrated PEDOT layer around 43 ± 5 µm and (C) stress-strain curves of the PTE film and PTE-PEDOT film swollen in PBS allowing to measure the Young's modulus of PTE and PTE-PEDOT films, and to calculate the Young's modulus of the PEDOT layer.

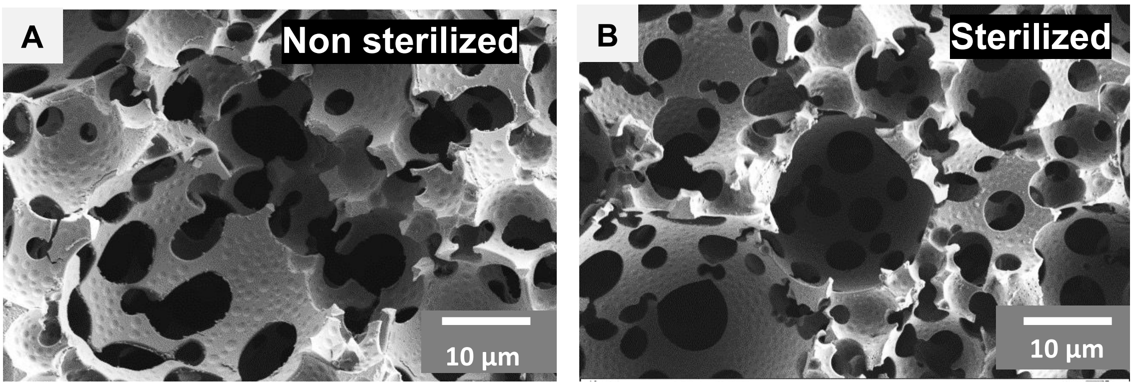

Figure S2. SEM images of polyHIPE/PEDOT before (A) and after the sterilization process via autoclave (B).

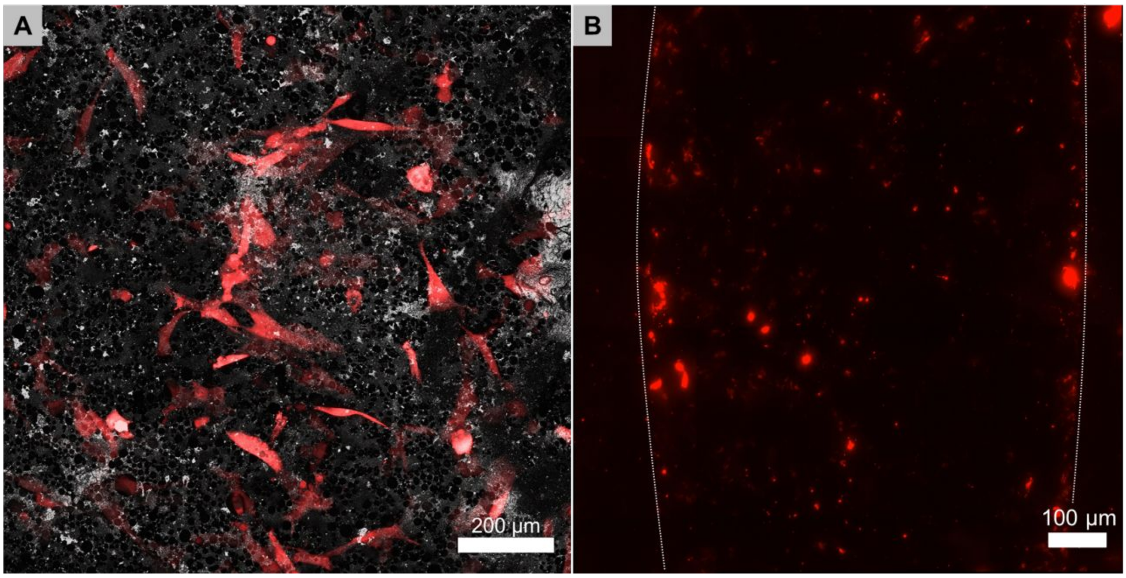

Figure S3. PolyHIPE/PEDOT scaffold (gray) seeded with 150,000 (A) and 500,000 (B) TTFLUOR HDF cells (red) after 3 days of incubation. Surface and first layer of the scaffold (A) and a cross-section of the scaffold (B) were acquired by fluorescence widefield microscopy. Cells were detected on surfaces; first layer and within the scaffold. White dotted line indicates the cross-section edges (B).

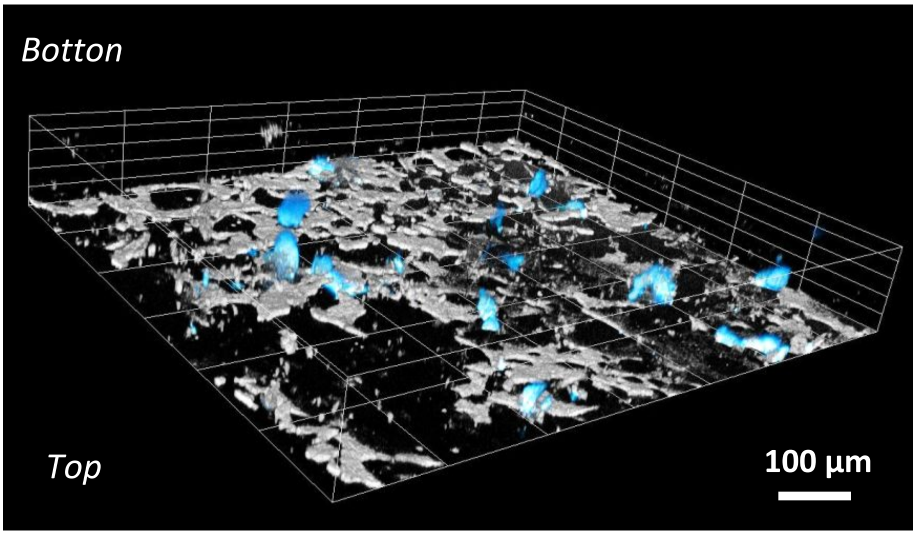

Figure S4. 3D representation of polyHIPE/PEDOT scaffold (confocal microscopy) incubated with BJ cells (incubation time 3h) 45 min after the trypsinization step. Nuclei DNA was stained with DAPI (blue).

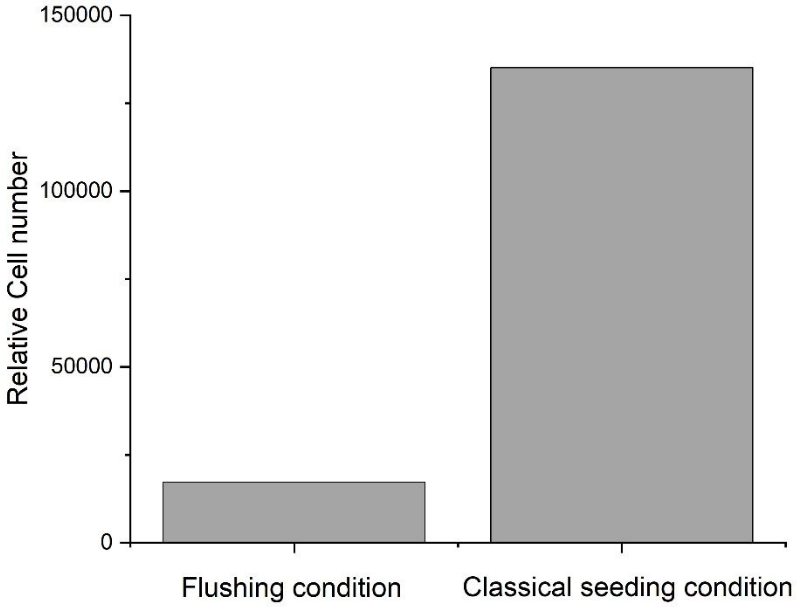

Figure S5. DNA quantification of Red TTFLUOR HDF cells seeded on polyHIPE/PEDOT scaffold 24h after flushing and removing cell suspension (left) or in classical seeding condition (right).

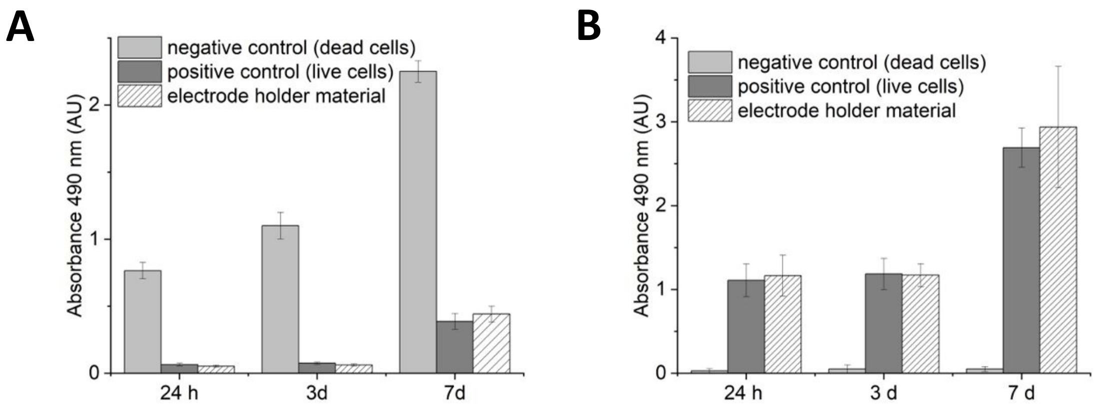

Figure S6. (A) LDH activity and (B) DNA content of BJ cells cultured on autoclaved 3D printing material (Smart Again ®) to evaluate cytotoxic effects of the electrode holder. Negative control (2D cell culture with Triton100X - dead cells) in gray, positive control (2D cell culture without treatment - live cells) in red and SmartAgain® (2D cell culture with treatment – SmartAgain®) in blue.

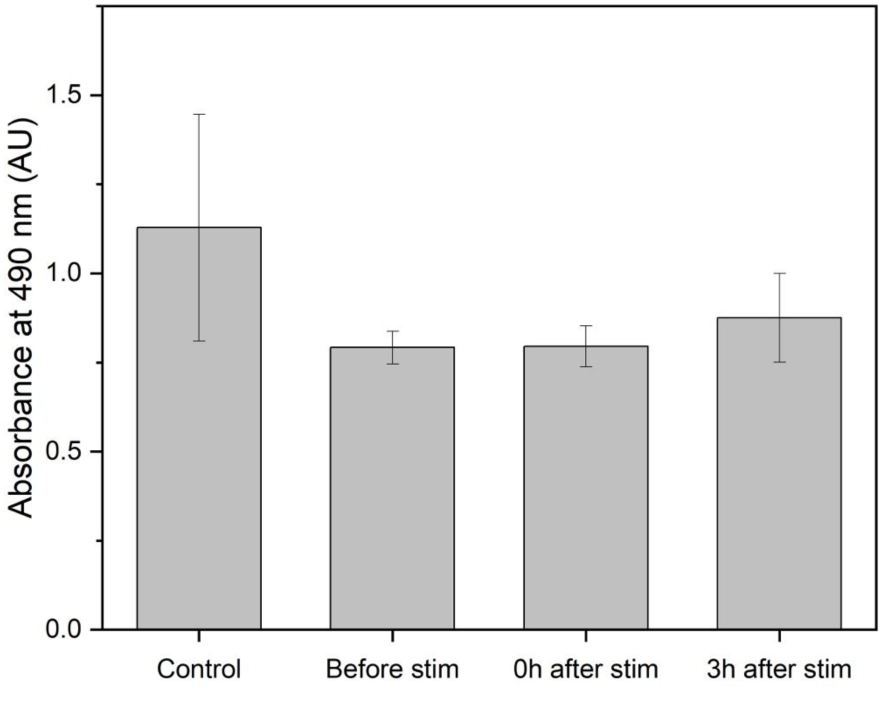

Figure S7. LDH activity before stimulation and 0 h or 3 h after a 10 min stimulation.

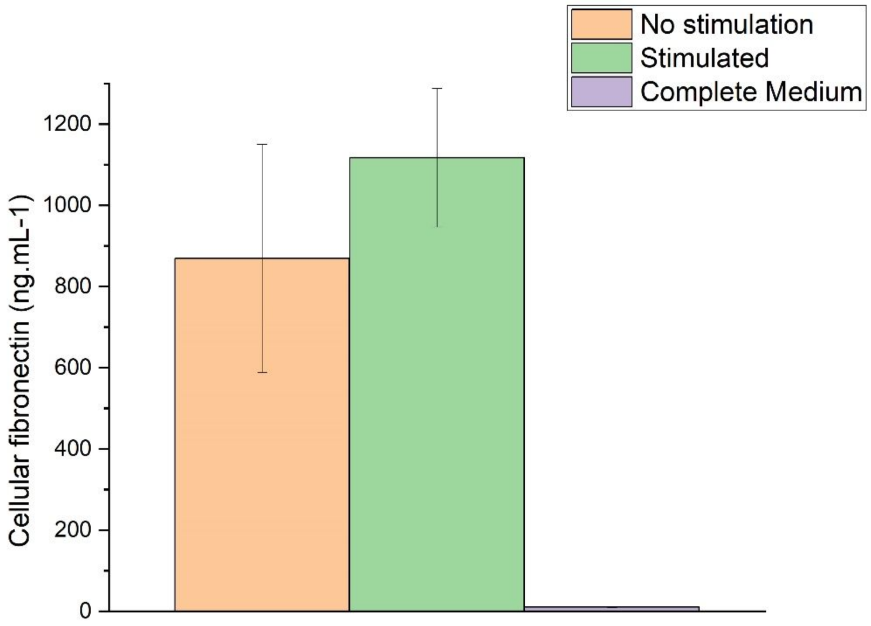

Figure S8. ELISA assay of secreted fibronectin from cells seeded on polyHIPE/PEDOT scaffold without any stimulation (orange), after 5 h of stimulation (100 cycles, 90 s per oxidation/reduction step, +/-1.7 V, green). Reference is complete media (DMEM, FBS 10%). No significative difference was seen between the two conditions. Complete cell culture media control confirmed the specificity of the ELISA assay, Fn from bovine serum was not recognized by human fibronectin primary antibody.

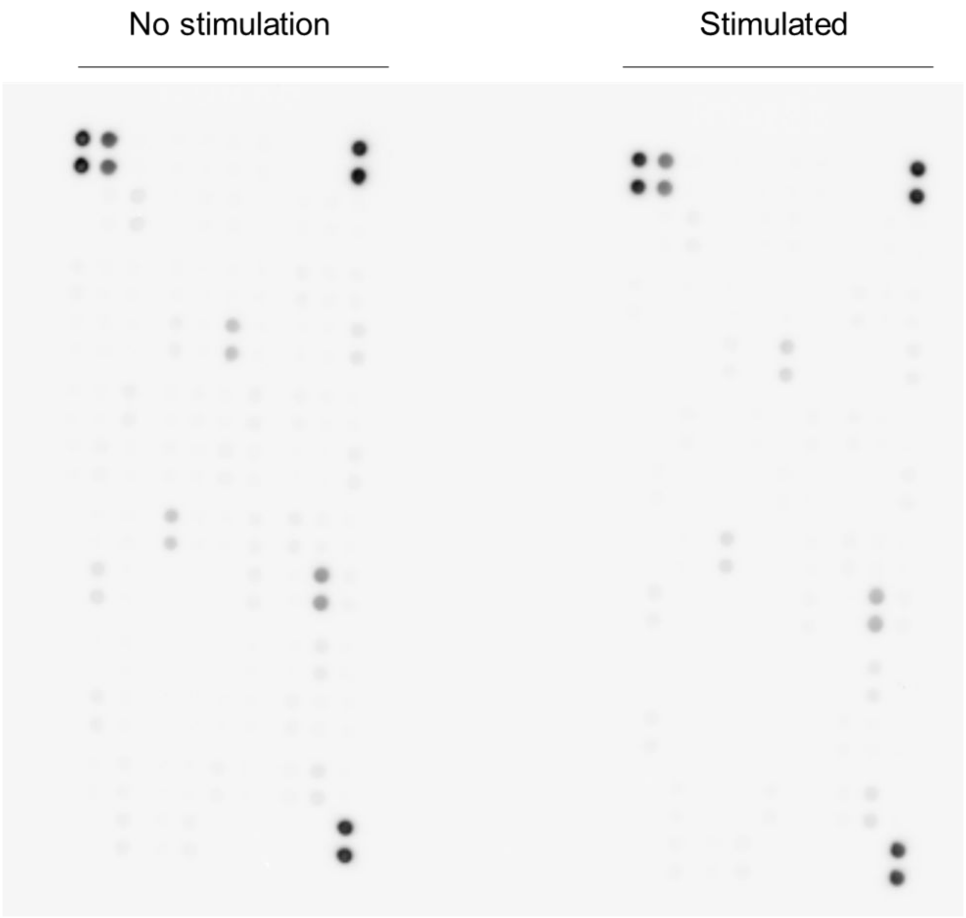

Figure S9. Cytokine array (Human XL Cytokine Array ARY022B, RnD systems) of cytokines released by BJ cells stimulated during 5 h on polyHIPE/PEDOT scaffolds (100 cycles, 90 s per oxidation/reduction step, +/-1.7 V). Unstimulated cells were also cultured on a polyHIPE/PEDOT scaffold for 5 h and were used as a reference.
